# Precision of slot widths and torque transmission of in-office 3D printed brackets: An in vitro study

**DOI:** 10.1007/s00056-023-00460-8

**Published:** 2023-03-02

**Authors:** Carolien A. J. Bauer, Mats Scheurer, Christoph Bourauel, J. Philippe Kretzer, Christoph J. Roser, Christopher J. Lux, Lutz D. Hodecker

**Affiliations:** 1 Poliklinik für Kieferorthopädie, Universitätsklinikum Heidelberg, Im Neuenheimer Feld 400, 69120 Heidelberg, Germany; 2 Klinik und Poliklinik für Mund‑, Kiefer‑, Gesichtschirurgie, Universitätsklinikum Heidelberg, Im Neuenheimer Feld 400, 69120 Heidelberg, Germany; 3 Oralmedizinische Technologie, Zentrum für Zahn‑, Mund- und Kieferheilkunde, Universitätsklinikum Bonn, Welschnonnenstr. 17, 53111 Bonn, Germany; 4 Labor für Biomechanik und Implantatforschung, Klinik für Orthopädie, Universitätsklinikum Heidelberg, Schlierbacher Landstraße 200a, 69118 Heidelberg, Germany

**Keywords:** Stereolithography process, Individual orthodontic braces, Slot precision, Manufacturing precision, Customized appliance, Stereolithographie-Verfahren, Individualisierte kieferorthopädische Brackets, Slotpräzision, Fertigungspräzision, Individualisierte Apparatur

## Abstract

**Purpose:**

To investigate a novel in-office three-dimensionally (3D) printed polymer bracket regarding slot precision and torque transmission.

**Methods:**

Based on a 0.022″ bracket system, stereolithography was used to manufacture brackets (*N* = 30) from a high-performance polymer that met Medical Device Regulation (MDR) IIa requirements. Conventional metal and ceramic brackets were used for comparison. Slot precision was determined using calibrated plug gages. Torque transmission was measured after artificial aging. Palatal and vestibular crown torques were measured from 0 to 20° using titanium–molybdenum (T) and stainless steel (S) wires (0.019″ × 0.025″) in a biomechanical experimental setup. The Kruskal–Wallis test with post hoc test (Dunn–Bonferroni) was used for statistical analyses (significance level *p* < 0.05).

**Results:**

The slot sizes of all three bracket groups were within the tolerance range according to DIN 13996 (ceramic [C]: 0.581 ± 0.003 mm; metal [M]: 0.6 ± 0.005 mm; polymer [P]: 0.581 ± 0.010 mm). The maximum torque values of all bracket–arch combinations were above the clinically relevant range of 5–20 Nmm (PS: 30 ± 8.6 Nmm; PT: 27.8 ± 14.2 Nmm; CS: 24 ± 5.6 Nmm; CT: 19.9 ± 3.8 Nmm; MS: 21.4 ± 6.7 Nmm; MT: 16.7 ± 4.6 Nmm).

**Conclusions:**

The novel, in-office manufactured polymer bracket showed comparable results to established bracket materials regarding slot precision and torque transmission. Given its high individualization possibilities as well as enabling an entire in-house supply chain, the novel polymer brackets bear high potential of future usage for orthodontic appliances.

## Abstract (de)

**Ziel:**

Untersuchung eines neuartigen, im 3‑D-Druckverfahren hergestellten Polymerbrackets im Hinblick auf Slotpräzision und Torqueübertragung.

**Methode:**

Basierend auf einem 0,022″-Bracketsystem wurden im Stereolithographie-Verfahren Brackets aus einem Hochleistungspolymer hergestellt (*n* = 30), die Anforderungen gemäß dem MDR-IIa (Medical Device Regulation) erfüllen. Als Vergleichsgruppe dienten konventionelle Metall- und Keramikbrackets. Die Slotpräzision der Brackets wurde mit Hilfe kalibrierter Lehrdorne überprüft, die Torqueübertragung unter dem Einfluss einer künstlichen Alterung gemessen. Dabei wurde ein palatinaler und vestibulärer Kronentorque von 0 bis 20° in Kombination mit Titan-Molybdän(T)- und Edelstahl(S)-Drähten (0,019″ × 0,025″) in einem biomechanischen Versuchsaufbau simuliert und gemessen. Die statistische Auswertung erfolgte mittels Kruskal-Wallis-Test mit Post-hoc-Test (Dunn-Bonferroni; Signifikanzniveau: *p* < 0,05).

**Ergebnisse:**

Die Slotgrößen aller 3 Bracketgruppen lagen innerhalb des Toleranzbereichs nach DIN 13996 (Keramik [C]: 0,581 ± 0,003 mm; Metall [M]: 0,6 ± 0,005 mm; Polymer [P]: 0,581 ± 0,010 mm). Die maximalen Drehmomentwerte aller Bracket-Bogen-Kombinationen lagen über dem klinisch relevanten Bereich von 5–20 Nmm (PS: 30 ± 8,6 Nmm; PT: 27,8 ± 14,2 Nmm; CS: 24 ± 5,6 Nmm; CT: 19,9 ± 3,8 Nmm; MS: 21,4 ± 6,7 Nmm; MT: 16,7 ± 4,6 Nmm).

**Schlussfolgerungen:**

Das neuartige im 3‑D-Druckverfahren hergestellte Polymerbracket zeigte hinsichtlich der Slotpräzision und der Torquebelastung mit den von etablierten Bracketsystemen vergleichbare Ergebnisse. Aufgrund der hohen Individualisierbarkeit und der Möglichkeit einer kompletten In-office-Versorgungskette zeigen die neuartigen Polymerbrackets vielversprechendes Potenzial im Hinblick auf eine klinische Anwendung.

## Introduction

The demand for orthodontic appliances with a low impact on orofacial esthetics, especially in the anterior region, continues to be high due to increased societal pressure regarding self-optimization and perfection [1, 2]. Currently, practicing orthodontists have the choice between numerous esthetic multibracket appliances for fixed therapy, which differ in geometry, material composition, and treatment efficiency. Regarding therapy efficiency, the most relevant factors are torque efficacy, bracket wing stability, binding and notching, resistance to the intraoral environment, adhesion to the enamel, and the possibility of gentle removal from the enamel. Regarding esthetics, important factors include color, size, and color stability. Patients’ wish for almost invisible orthodontic appliances has led to the development of tooth-colored bracket materials, primarily made of ceramics, polymer materials or a combination of both [3, 4].

In contrast to lingual bracket systems, which have the best esthetic appearance and very effective torque, tooth-colored brackets can be integrated into the proven classic straight-wire concept [5–7]. Ceramic brackets are considered an established alternative to conventional metal brackets and are characterized by high stability and rigidity as well as a low tendency to deformation and discoloration [8, 9]. However, the hardness of the ceramic material can lead to an increased risk of enamel abrasion [10]. Further practical challenges can include problems regarding adhesive bonding to the enamel, high shear bond strength, high sliding resistance, high susceptibility to fracture and chipping during torque loading, and the time-consuming debonding procedure [11–13].

Brackets made of polymer material provide another esthetic alternative to metallic brackets. Polycarbonates are thermoplastics, which allow easy matching to the tooth color thanks to their transparent properties [14]. In addition, they have been shown to exhibit low sliding resistance [15–17]. However, given their poor stability and stiffness, resulting in permanent plastic deformation even at low force, polymer brackets are only rarely used in practice [18]. Testing of different material compositions of polycarbonate and polyurethane with inorganic fillers, such as glass fibers or ceramic particles, and/or a metal cover for the slot have also failed to meet clinical requirements [3, 4]. The effective torque of brackets made of these materials was continuously significantly lower than that of ceramic and metal brackets [8, 14, 19]. In addition, their color stability was only moderate [20, 21]. This was also observed for novel resins used in three-dimensional (3D) printing [22].

The material-specific shortcomings of both ceramic and current polymeric brackets have led to efforts in developing a more suitable material for tooth-colored brackets. With 3D printing being on the rise in dentistry, the first steps to produce in-office printed brackets have already been published [23–26]. A novel high-performance resin, Permanent Crown Resin ([PCR], Formlabs Inc., Somerville, MA, USA), approved for the fabrication of permanent dental crowns has recently become available for 3D stereolithographic printing (SLA). If PCR meets the requirements for prosthetic crowns, we hypothesized that it might also be suitable as a bracket material. The objective of this study was to investigate the material-specific characteristics of an in-office manufactured PCR bracket in terms of fabrication quality, fabrication precision, and torque transmission after simulated artificial aging in a thermocycler. Our specific interest was the effective torque of the PCR brackets, the specific dimension in which all previous polymer materials have performed poorly. Such in-office manufactured PCR brackets could offer an alternative to conventional tooth-colored bracket systems, as they provide advantages in terms of personalization regarding bracket color, bracket prescription, and bracket base.

## Materials and methods

### Bracket systems

Two established conventional, prefabricated metal and ceramic bracket systems and a novel in-office manufactured bracket system made of PCR were investigated. In this study, in-office refers to the production in one’s own dental practice or clinic. The prefabricated brackets were purchased from one manufacturer (discovery® made of metal and discovery® pearl made of high-purity polycrystalline aluminum oxide ceramic by Dentaurum GmbH & Co. KG, Ispringen, Germany). According to the manufacturer, these brackets belong to the 0.022″ (0.5566 mm) slot system. The in-office manufactured PCR brackets were produced using SLA printing and the high-performance polymer Permanent Crown Resin A2 based on an edgewise semi-twin bracket (Table 1).

**Table 1:** Different bracket types, manufacturer’s data, and mechanical properties of the tested materials [27]. As there is no data available regarding the mechanical properties of discovery® pearl and discovery® smart, the information provided here is based on the material class of polycrystalline aluminum oxide ceramics and 1.4404 stainless steel [28–30] Verschiedene Brackettypen, Herstellerangaben und mechanische Eigenschaften der getesteten Materialien [27]. Die Daten zu den mechanischen Eigenschaften von discovery® pearl und discovery® smart beziehen sich hier auf Vergleichswerte der Materialklasse der polykristallinen Aluminiumoxidkeramiken und des Edelstahls 1.4404 [28–30]

Bracket material	Manufacturer	Slot dimension (mm)	Configuration	Production method	Flexural strength (MPa)	Flexural modulus (GPa)
1.4404 stainless steel	Dentaurum	0.5566	True-twin	MIM	500–700	196
Polycrystalline aluminum oxide ceramic	Dentaurum	0.5566	True-twin	CIM	600	380
Permanent Crown Resin	In-office	0.5566	Semi-twin	SLA	119–144	4.37–4.69

*MIM* metal injection molding, *CIM* ceramic injection molding, *SLA* 3D stereolithographic printing

### Manufacturing the in-office PCR bracket

**Fig. 1:**
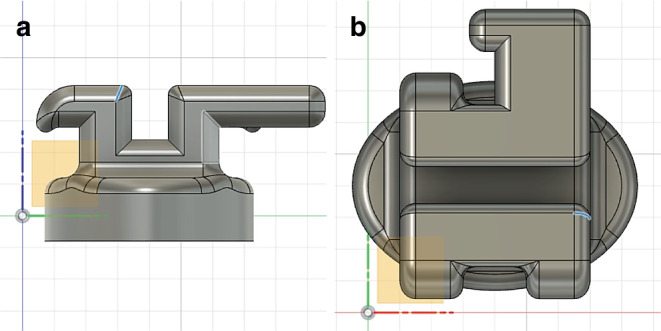
In-office manufactured Permanent Crown Resin (PCR) bracket based on a semi-twin bracket design. **a** side view; **b** top view; software used: Fusion 360® CAD (computer-aided design) software platform In office hergestelltes PCR(Permanent Crown Resin)-Bracket auf der Basis eines Semi-Twin-Bracket-Designs. **a** Ansicht Seite; **b** Ansicht oben; verwendete Software: Fusion 360® CAD(„computer-aided design“)-Softwareplattform

The PCR brackets were manufactured using the Formlabs 3b SLA printer (Formlabs Inc., Somerville, MA, USA). Figure 1 shows the bracket geometry, which was designed using the Fusion 360® CAD (computer-aided design) software platform (Autodesk GmbH, Munich, Germany). This software can be used for the creation of individual designs in terms of form, fit, and function. The bracket sketch was based on a classic bracket design consisting of a bracket trunk, bracket base, bracket wings, and a bracket slot. Programming of the bracket in terms of in/out, torque and tip was not relevant for the present study but would be possible for future research.

After designing the bracket geometry, the STL data set was transferred to the printer-specific software PreForm (Formlabs Inc., Somerville, MA, USA). Using PreForm, the brackets were positioned on the printer platform. After aligning the bracket slots according to the z‑axis, additional support structures were positioned without affecting the sensitive area of the bracket slots and allowing complete printing of the geometries. Such support structures are used to map even complex geometries in the SLA process.

**Fig. 2:**
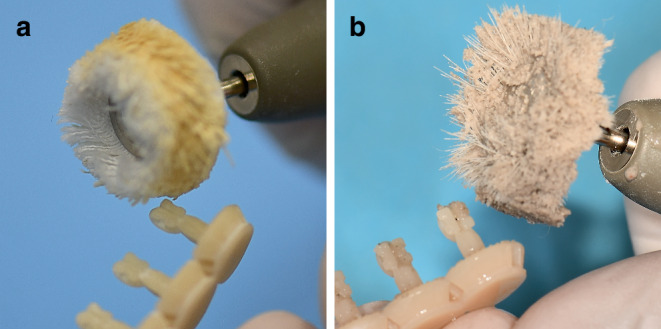
Manual polishing of the in-office manufactured Permanent Crown Resin (PCR) brackets with **a** pumice stone and **b** polishing paste Manuelle Politur der „in office“ hergestellten PCR(Permanent Crown Resin)-Brackets mit **a** Bimsstein und **b** Polierpaste

PCR is a photosensitive resin based on the esterification product of a phenol and a methacrylic acid. Silanized dental glass (particle size: 0.7 µm) is added as an inorganic filler at a mass percent of 30–50. The polymer cures at 385 and 405 nm. The highest resolution of the printer is 25 µm in the xy direction. For the PCR, a supported print resolution of 50 µm is listed and it is certified for appropriate clinical use meeting Medical Device Regulation class IIa requirements [27]. Considering the manufacturing tolerance of the 3D printer, a 5% oversizing in the slot height (incisal–gingival) was set. After the printing process, the brackets were released from the platform. Manual postprocessing was performed according to the protocol specified by the manufacturer: First, the brackets were placed in a 99% isopropyl alcohol solution in the Form Wash (Formlabs Inc., Somerville, MA, USA) for 3 s to remove excess resin. After 30 min of air drying, the brackets were checked for remaining resin and cleaned manually. Postpolymerization followed in the Form Cure curing chamber (Formlabs Inc., Somerville, MA, USA) at 60 °C for 20 min and ultraviolet light (405 nm). Afterwards, the support structures were removed manually; the brackets roughly finished and cured again at 60 °C for 20 min. The final curing was followed by polishing with pumice stone and polishing paste and cleaning with distilled water (Fig. 2).

### Manufacturing precision

**Fig. 3:**
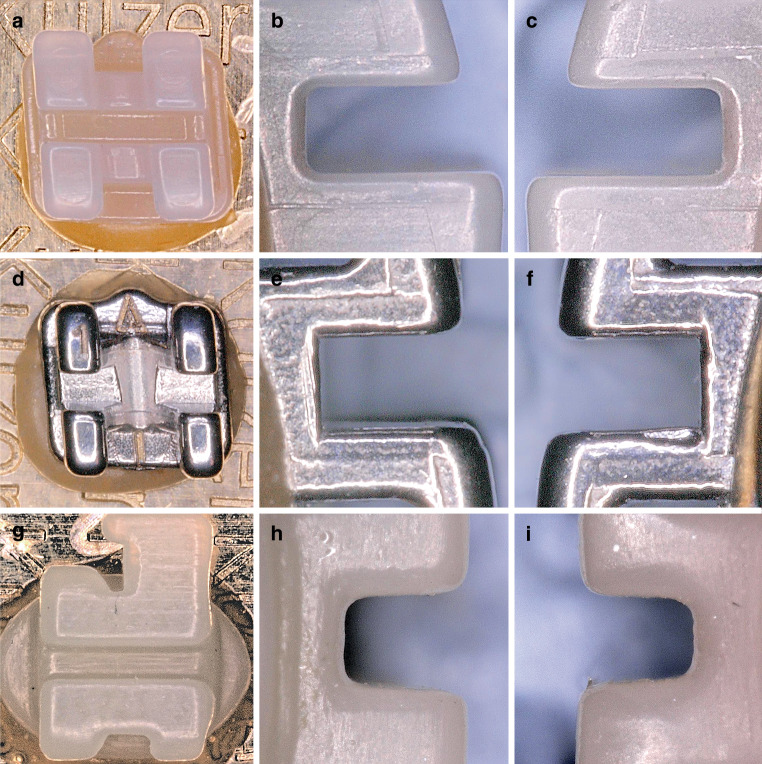
Brackets before artificial aging and torque loading from frontal (magnification ×100), distal and mesial (magnification ×150). **a–c** ceramic bracket discovery® pearl, **d–f** metal bracket discovery®, **g–i** in-office manufactured Permanent Crown Resin (PCR) bracket; light microscope images Brackets vor künstlicher Alterung und Torquebelastung von frontal (Vergr. 100:1), distal und mesial (Vergr. 150:1). **a–c** Keramikbracket discovery® pearl, **d–f** Metallbracket discovery®, **g–i** „in office“ hergestelltes PCR(Permanent Crown Resin)-Bracket; lichtmikroskopische Aufnahmen

**Fig. 4:**
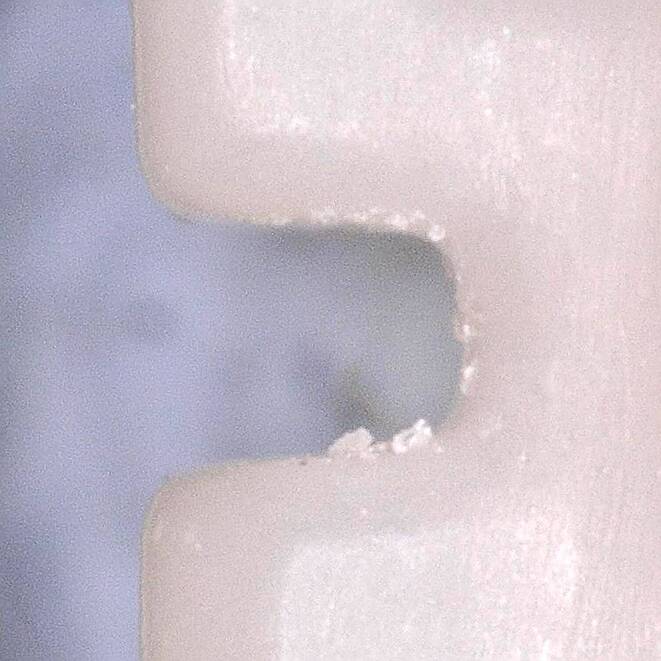
Residue on an in-office manufactured Permanent Crown Resin (PCR) bracket slot from the mesial point of view; light microscope image (magnification ×150) Rückstände auf einem in office hergestellten PCR(Permanent Crown Resin)-Bracketslot aus mesialer Sicht; lichtmikroskopische Aufnahme, Vergr. 150:1

**Fig. 5:**
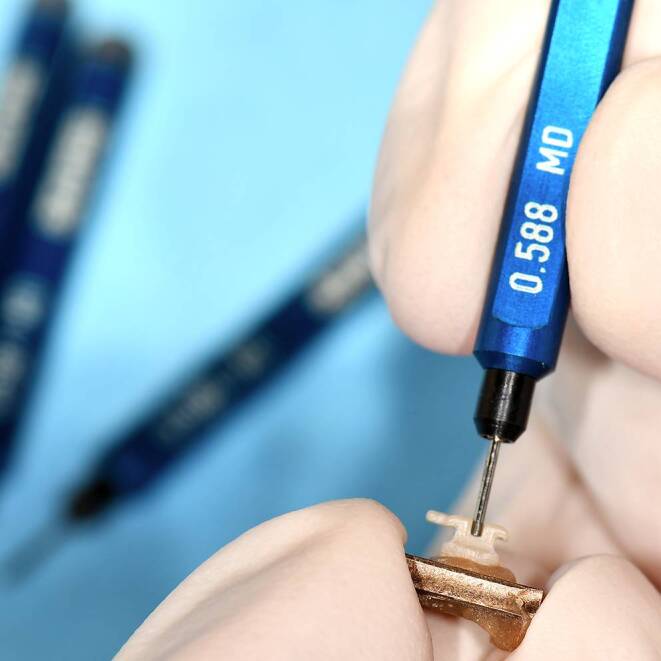
Measurements of the slot dimensions for metal, ceramic, and Permanent Crown Resin (PCR) brackets with hard-metal single handle plug gages with rounded tips Messungen der Slotdimensionen für Metall‑, Keramik- und PCR(Permanent Crown Resin)-Brackets mit Hartmetall-Lehrdornen mit abgerundeten Spitzen

To ensure correct orientation, all 90 brackets (30 PCR, 30 ceramic, 30 metal) were bonded to metal plates (5 mm × 5 mm) with a positioning aid (DeguDent GmbH, Hanau-Wolfgang, Germany) [17]. Visual inspection of the brackets was performed using a Keyence® VHX500 digital microscope (Keyence Corporation, Osaka, Japan; Fig. 3). Material defects and inaccuracies, especially in the slot area (frontal, mesial, and distal) were documented (Fig. 4). Visual inspection was repeated after the torque measurements had been performed. Slot sizes were measured from 0.556 to 0.612 mm in intervals of 0.002 mm at the entrance and base of the slot using specially manufactured calibrated hard-metal single handle plug gages with rounded tips with an accuracy of ± 0.0004 mm (Azurea Jauges SA, Belprahon, Switzerland) in accordance with ISO 17025 [31]. This range represents the permitted deviations according to DIN 13996 of up to 10% enlargement for a slot size of 0.022″ [32]. The determination of the slot size by the plug gages was carried out in ascending order from the smallest size to the largest possible size (Fig. 5). The inserted arch wires were measured with a digital micrometer IP65 0–25 mm (Mitutoyo IP65, Mitutoyo, Kawasaki, Japan) allowing for measurement with a deviation of 1 μm according to a previously published protocol [33].

### Artificial aging process and torque measurement

**Fig. 6:**
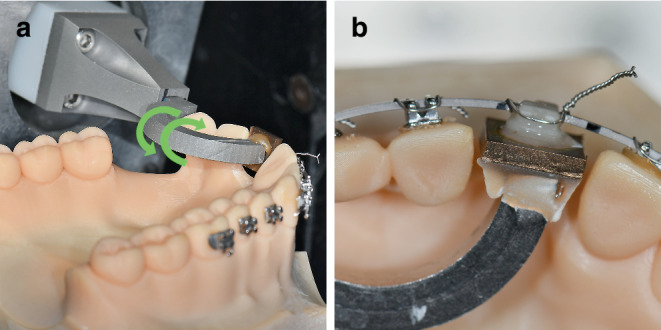
Orthodontic Measurement and Simulation System (OMSS) measurements: **a** fixed test model with ligated test arch wire on the positioning table. The guide arm with the attached test bracket is connected to the torque–force sensor of the measuring table. During the rotation of the guide arm the effective torque is measured. The *green arrows* visualize the movement for the vestibular and palatal crown torques, **b** test model with ligated arch wire and test bracket on the guide arm are connected via a wire ligature OMSS(Orthodontic Measurement and Simulation System)-Messungen: **a** fixiertes Testmodell mit einligiertem Bogen auf dem Positioniertisch. Der Führungsarm mit dem angebrachten Prüfbügel ist mit dem Drehmoment-Kraft-Sensor des Messtisches verbunden. Während der Drehung des Führungsarms wird das effektive Drehmoment gemessen. Die *grünen Pfeile* visualisieren die Bewegung für die vestibulären und palatinalen Kronenmomente. **b** Testmodell mit einligiertem Bogen und Testbracket am Führungsarm sind über eine Drahtligatur verbunden

To simulate aging, the brackets were subjected to thermocycling (+ 5 °C and + 55 °C for a total of 5000 cycles and storage in water at + 37 °C for 1 week), simulating a therapy of one year according to ISO 10477 [34]. Using the Orthodontic Measurement and Simulation System (OMSS) at the University of Bonn, Germany, the simulation of a palatal and vestibular crown torque on an upper central incisor (1.1) was carried out in the range of 0 to 20° with the three different bracket materials (Fig. 6). The OMSS consists of two measuring stages, both of which can be positioned over six axes and can record all force–torque vectors via a computer-aided 3D force–torque sensor. This enables examination of specific orthodontic biomechanical issues by simulating orthodontic tooth movements [35, 36]. As a working model, an upper jaw model from Frasaco (Frasaco GmbH, Tettnang, Germany) was mapped optically, the working space of the guide arm was digitally reduced, and 3D printed using model resin V2 (Formlabs Inc., Somerville, MA, USA). Additional brackets were passively bonded to the adjacent teeth of the working model and the orthodontic arch wire was ligated without tension using wire ligatures (remanium® preformed ligature; 0.01 inch; Dentaurum GmbH & Co. KG, Ispringen, Germany). Tooth 1.1 was replaced with a test bracket, which was previously bonded to a guide arm using a positioning key. The guide arm was then connected to the force/torque sensor. The test bracket was attached to the arch wire with a wire ligature so that it rested tension-free in the bracket–arch wire complex. The movement of the test bracket in terms of vestibular and palatal crown torques from 0 to 20° was first calculated by a mathematical model and measured in 0.5° increments using the stepping motor-driven positioning table. The resulting torque at the bracket was measured with an accuracy of 0.5 Nmm. After each simulation cycle, the bracket returned to its initial position in 0.5° steps. During this process, all load and unload positions were captured by the software. As part of the torque simulation, 160 measured values in Nmm were captured per cycle and processed using Microsoft Excel (Microsoft Corporation, Redmond, WA, USA).

### Statistical evaluation

The measurements of the slot dimensions in the gingival–incisal direction as well as the measurements of the palatal and vestibular crown torque up to 20° per bracket–arch combination were descriptively analyzed. The nonparametric Kruskal–Wallis test with a subsequent Dunn–Bonferroni post hoc test was performed to examine the differences between the maximum torque values at 20° rotation between the in-office manufactured PCR brackets and the control groups with ceramic and metal brackets (significance level: *p* < 0.05). All statistical analyses were performed using IBM SPSS Statistics version 28.0.1.0 statistical software for Windows (IBM, Armonk, NY, USA).

## Results

### Manufacturing quality

**Fig. 7:**
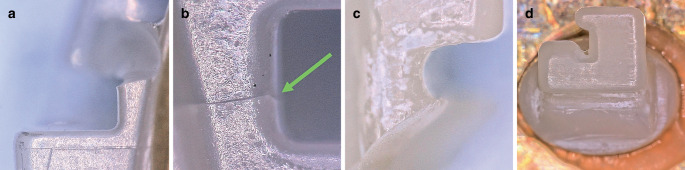
Bracket damages after artificial aging and torque loading on **a,** **b** discovery® pearl, **c,** **d** in-office manufactured Permanent Crown Resin (PCR) brackets: **a** Mesial view of the slot, broken bracket wing, magnification ×150, **b** distal view of slot bottom, *green arrow* points to fracture line, magnification ×200, **c** distal view of the slot, broken bracket wings, magnification ×150, **d** frontal view of the slot, broken slot wall, magnification ×100; light microscope images Bracketschäden nach künstlicher Alterung und Torquebelastung. **a,** **b** discovery® pearl, **c,** **d** „in office“ hergestelltes PCR(Permanent Crown Resin)-Bracket **a** mesiale Ansicht des Slots, gebrochener Bracketflügel, Vergr. 150:1, **b** distale Ansicht des Slotbodens, *grüner Pfeil* zeigt auf Frakturlinie, Vergr. 200:1, **c** distale Ansicht des Slots, gebrochene Bracketflügel, Vergr. 150:1, **d** frontale Ansicht des Slots, gebrochene Slotwand, Vergr. 100:1; lichtmikroskopische Aufnahmen

During the first visual inspection to assess the manufacturing quality of the brackets (metal, ceramic, PCR) material residues were observed on certain parts of the in-office manufactured PCR brackets. In addition, irregularities were visible regarding the polishing quality and in the bracket slot. The metal brackets showed notches and furrows. None of the materials displayed sharp edges. During the second visual inspection after artificial aging and torque measurements, slot widening was observed in the slot walls of the in-office manufactured PCR brackets. In one bracket of this material class, two bracket wings had broken off. For the metal brackets there were no changes compared to the initial observations. Bracket wings were partially chipped off in nine ceramic brackets. Figure 7 shows representative images of the damaged brackets.

### Manufacturing precision

**Fig. 8:**
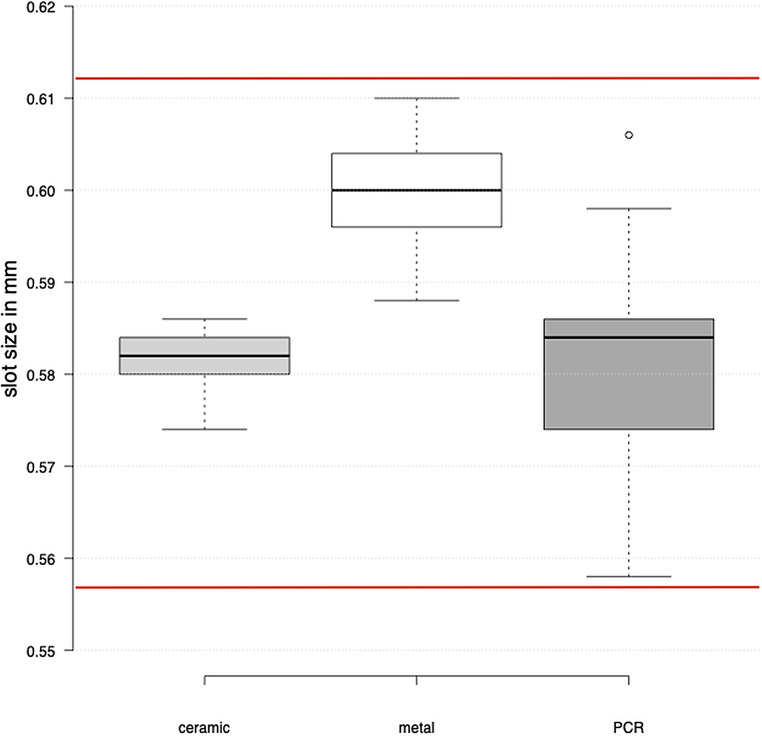
Slot sizes (in mm) of all bracket materials (ceramic, metal, Permanent Crown Resin [PCR]) for a 0.022″ system; box and whisker plot; *red lines* indicate the limits permitted according to DIN 13996 (*upper line*: 0.6120 mm; *lower line*: 0.5566 mm) [32] Slotgrößen (in mm) aller Bracketmaterialien (Keramik, Metall, PCR [Permanent Crown Resin]) für ein 0,022″-System; Box- und Whisker-Plot; die *roten Linien* kennzeichnen die nach DIN 13996 zulässigen Grenzen (*obere Linie*: 0,6120 mm; *untere Linie*: 0,5566 mm; [32])

The manual measurements of the bracket slot widths with the plug gages showed that all material groups were within the corresponding DIN 13996 regarding “Dimensions for arch wires and attachments for orthodontic appliances” (Fig. 8). The three material groups displayed notable differences in the slot widths (Table 2). The deviation from the nominal size of a 0.022″ (0.5566 mm) bracket system was the lowest for the ceramic brackets with a mean slot width (mean, SD) of 0.581 ± 0.003 mm. The highest deviation in slot dimension were observed for the metal brackets. The mean slot width in this group was 0.600 ± 0.005 mm. The mean slot width for the PCR brackets was 0.581 ± 0.010 mm at the slot bottom. A difference of + 0.003 ± 0.000 mm (+ 3%) from the slot entrance to the slot bottom was measured. In the other two material groups, no difference could be measured between the slot entrance and bottom. The variance of slot widths was higher for the PCR brackets (SD 0.010 mm) than for the ceramic (SD 0.003 mm) and metal (SD 0.005 mm) brackets. The measurements of the arch wire thickness with the digital micrometer showed a mean value of 0.483 mm for SS (stainless steel) and TMA (titanium–molybdenum) according to DIN 13996 [32].

**Table 2:** Descriptive statistics of the mean slot sizes of the three bracket materials (ceramic, metal, PCR). Only the PCR brackets showed differences between the slot entrance and slot bottom. *PCR* Permanent Crown Resin Deskriptive Statistik der mittleren Slotgrößen der 3 Bracketmaterialien (Keramik, Metall, PCR). Nur bei den PCR-Brackets wies der Slot Unterschiede zwischen dem Slot-Eingang und dem Slot-Boden auf. *PCR* Permanent Crown Resin

		*N*	Mean (mm)	SD (mm)	Minimum (mm)	Maximum (mm)
*Ceramic*		30	0.581	0.003	0.574	0.586
*Metal*		30	0.600	0.005	0.588	0.610
*PCR*	Bottom	30	0.581	0.010	0.558	0.606
	Entrance	30	0.586	0.007	0.572	0.610

*SD* standard deviation, *PCR* Permanent Crown Resin

### Effective torque

**Fig. 9:**
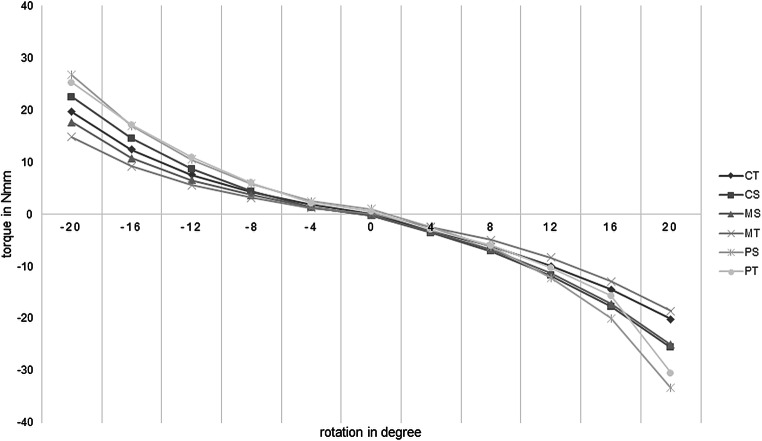
Line diagram of the mean values of the measured torques (Nmm) of the simulated palatal and vestibular crown torques (°) for each bracket–arch wire combination. *PCR* Permanent Crown Resin, *PS* PCR–stainless steel, *PT* PCR–titanium-molybdenum, *CS* ceramic–stainless steel, *CT* ceramic–titanium-molybdenum, *MS* metal–stainless steel, *MT* metal-titanium-molybdenum Liniendiagramm der Mittelwerte der gemessenen Drehmomente (Nmm) der simulierten palatinalen und vestibulären Kronenmomente (in Grad) für jede Bracket-Bogen-Draht-Kombination. *PCR* Permanent Crown Resin, *PS* PCR-Edelstahl, *PT* PCR-Titan-Molybdän, *CS* Keramik-Edelstahl, *CT* Keramik-Titan-Molybdän, *MS* Metall–Edelstahl, *MT* Metall–Titan-Molybdän

A crown torque of ± 20° was simulated using the OMSS. The effective torque values were measured for each bracket–arch wire combination (CS = ceramic–stainless steel; CT = ceramic–titanium-molybdenum; MS = metal–stainless steel; MT = metal–titanium-molybdenum; PS = PCR–stainless steel; PT = PCR–titanium-molybdenum). The data were adjusted for initial negative values. The values for the broken brackets were removed. Figure 9 shows the measured values for the simulation of palatal and vestibular crown torque of up to 20° in all bracket–arch wire combinations. PS displayed the steepest rise in torque and the highest torque values, followed by PT. MT displayed the shallowest slope and the lowest torque values. Overall, the maximum torque values of all bracket–wire combinations showed a comparable trend.

**Fig. 10:**
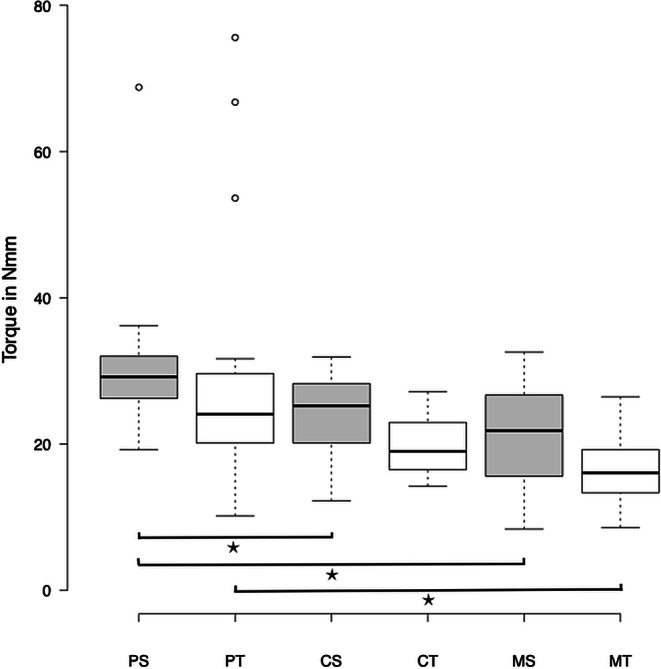
Box and whisker plot of the maximum torque values of all bracket–arch wire combinations. *Asterisks* indicate differences that reached significance (*p* ≤ 0.05, Kruskal–Wallis test and post hoc Dunn–Bonferroni tests). *PCR* Permanent Crown Resin, *PS* PCR-stainless steel, *PT* PCR-titanium-molybdenum, *CS* ceramic-stainless steel, *CT* ceramic-titanium-molybdenum, *MS* metal-stainless steel, *MT* metal-titanium-molybdenum Box- und Whisker-Plot der maximalen Drehmomentwerte aller Bracket-Bogen-Draht-Kombinationen. Die *Sternchen* kennzeichnen signifikante Unterschiede (*p* ≤ 0,05, Kruskal-Wallis-Test und Post-hoc-Dunn-Bonferroni-Tests). *PCR* Permanent Crown Resin, *PS* PCR-Edelstahl, *PT* PCR-Titan-Molybdän, *CS* Keramik-Edelstahl, *CT* Keramik-Titan-Molybdän, *MS* Metall-Edelstahl, *MT* Metall-Titan-Molybdän

The Kruskal–Wallis test was performed in order to test for inequality in maximum torque transmission between the three bracket materials and the two arch wire materials. Results indicate significant differences in effective torque between the bracket materials for the bracket–SS combinations and bracket–TMA combinations (bracket–SS: χ^2^ = 19.439, *p* ≤ 0.001; bracket–TMA: χ^2^ = 23.560, *p* ≤ 0.001). The post hoc tests (Dunn–Bonferroni tests) showed significant differences between the PS/MS, PS/CS, and PT/MT groups (PS/MS: *p* ≤ 0.001; PS/CS: *p* = 0.015; PT/MT: *p* ≤ 0.001), indicating significant differences in torque transmission between the tested bracket–arch combinations. For PS/MS and PT/MT, the results indicate a strong effect (PS/MS: r = 0.57; PT/MT: r = 0.56). For PS/CS, the results indicate a medium effect (r = 0.38). No significant differences were observed for the fourth group PT/CT (*p* = 0.066). Nine ceramic and one PCR bracket were fractured during testing. Table 3 shows the descriptive values for the maximum torques of all bracket–arch wire combinations. Figure 10 shows a box and whisker plot of the recorded torques at maximum rotation (20°).

**Table 3:** Descriptive statistics of the maximum torque values (Nmm) of all bracket–arch wire combinations Deskriptive Statistik der maximalen Drehmomentwerte (Nmm) aller Bracket-Bogendraht-Kombinationen

	*N*	Minimum (Nmm)	Maximum (Nmm)	Mean (Nmm)	SD (Nmm)
*PS*	28	19.2	68.8	30.0	8.6
*PT*	30	10.2	75.6	27.8	14.2
*CS*	24	12.2	31.9	24.0	5.6
*CT*	18	14.3	27.2	19.9	3.8
*MS*	30	8.4	32.6	21.4	6.7
*MT*	30	8.6	26.5	16.7	4.6

*SD* Standard deviation, *PCR* Permanent Crown Resin, *PS* PCR–stainless steel, *PT* PCR–titanium-molybdenum, *CS* ceramic–stainless steel, *CT* ceramic–titanium-molybdenum, *MS* metal–stainless steel, *MT* metal–titanium-molybdenum

## Discussion

In the present study, a novel high-performance resin, which has been shown to be suitable for dental crown fabrication, was transferred to orthodontic brackets manufacturing and compared with conventional bracket materials (ceramic, metal) regarding slot precision and effective torque [27]. Results showed comparable performance of the novel PCR bracket to the established bracket materials regarding both metrics.

To detect possible visible defects, a visual inspection of the brackets was performed under a digital microscope. This step served as an initial evaluation of the manufacturing quality. Special attention was paid to the implementation of the digital bracket design especially regarding rounded edges, excess resin residues, and polishing defects. Of course, this methodology only has limited validity regarding biomechanical properties; thus, further tests were carried out [17].

In accordance with previous studies, the dimension of the used wires was measured with a digital micrometer [33]. The manufacturing precision in the slot area was determined with specially calibrated plug gages. Based on ISO certification, the plug gages were selected with an accuracy of ± 0.0004 mm and were, thus, considered suitable for determining slot sizes [31]. It should be noted that this procedure does not allow precise three-dimensional slot measurement. However, in the present study the slot size measurements were only relevant for the subsequent interpretation of the torque values; thus, the comparability of slot sizes between the material groups suffices. Further studies will be necessary to verify the exact morphological characteristics of the PCR slots, e.g., by using established methods such as visual measurement in a digital optical microscope, machine measurement using specially manufactured inspection systems, or with the aid of a micro-CT (computed tomography) [14, 17, 37–39].

The obtained results of the slot measurements and wire dimension were all within the range specified by the DIN 13996 [32]. The observed oversizing of the slot heights of the conventional bracket groups was expected and is consistent with previous literature [37, 38, 40, 41]. For the PCR brackets, the slot size was set at 5% above the target of 0.022″ to meet the required precision of 50 µm of the PCR material for SLA printing. Surprisingly, the print precision in the slot size area exhibited a deviation of only 10 µm, showing higher precision than anticipated. In contrast to the metal and ceramic brackets, the PCR brackets displayed a difference in the slot height with slot entrance being 3% larger than the slot bottom on average. Furthermore, the PCR brackets showed a higher variance in slot sizes than the other two material groups. A possible explanation for these differences could be the differences in manufacturing of the bracket groups. The ceramic brackets used in the present study were manufactured using the ceramic injection molding process (CIM), the metal brackets using the metal injection molding process (MIM), each procedure followed by a mechanical finishing process. Both are standardized mechanical processes that ensure a high degree of precision [42, 43]. The resolution of 3D printing and manual post-processing can be assumed to have been the causes for the higher variance of the measurements of the PCR brackets.

Polymers have the property of absorbing water and therefore swell due to the water absorption in the intraoral environment [4, 21, 44]. To mimic real life behavior of the material, artificial aging according to ISO 10477 was performed [34]. It should be noted that thermocycling only conditionally mirrors the real clinical situation and is limited regarding influences such as food intake and the associated pH fluctuation, chewing and abrasion of surfaces, home and professional tooth cleaning, and mineral concentration in saliva. Further research is necessary to determine the influence of these factors on material properties.

The OMSS was utilized to determine torque movements. This is in line with current literature, which deems the OMSS suitable for in vitro studies of biomechanical issues related to different tooth movements in all spatial planes [4, 45–48]. In line with previous literature, torque transmission was higher for steel arch wires than for TMA arch wires. Reasons for this may be the different E‑moduli and/or the different flexural strengths [49, 50].

The obtained mean torque values of the PCR brackets were generally comparable to, and at times slightly higher than those of the reference groups. These results differ from the results of previous studies of polymer brackets with a wide variety of polymer compositions regarding torque stability. In these studies, the polymer brackets never achieved the effective torque values of the ceramic and/or metal reference groups, due to deformation and slot expansion [4, 8, 14, 47, 51–53]. Surprisingly, the small deformations of the bracket slots of the PCR brackets after torque loading, as seen during the visual inspection, had no effect on the maximum torque values in the present study. Effects on the tip and friction behavior have not been investigated. One reason for the good performance of the novel high-performance polymer for bracket fabrication could be its special composition [27]. Another reason could be the novel manufacturing 3D-printing process.

The variance of torque values was higher for the PCR brackets than for the reference groups. This is probably due to the higher variation in slot width of the PCR brackets resulting in higher variation of slot play. Slot play describes the empty space between the bracket and the arch wire caused by the combination of oversized bracket slots and/or undersized arch wire cross sections. This causes torque to be initiated only after the arch wire has undergone a certain amount of torsion [7, 40, 41]. For example, in a study by Joch et al., examinations of self-ligating and conventional brackets revealed actual high slot play due to oversized slots [33].

In general, the effective torque values measured in the present study far exceeded those of clinical relevance. In daily practice, the effective applied torques are usually between 5 and 20 Nmm [13, 35–37]. Our simulation in the OMSS provided initial evidence that the in-office manufactured PCR brackets can meet these clinical requirements. Further in vivo research is necessary to validate these findings.

That the novel PCR brackets could be a promising alternative to established material groups in clinical practice is further supported by the fact that only one of 30 PCR brackets broke during torque loading in the present study. In contrast, 9 of 30 ceramic brackets shattered. One explanation could be the differences of these materials regarding their mechanical properties. In the study by Grzebieluch et al., the material properties of novel polymers, including PCR, were investigated [23, 27]. The reported values of the flexural modulus (4.37–4.69 GPa) were significantly lower than those of polycrystalline alumina oxide ceramics [30]. The relatively low flexural modulus could explain the lower fracture susceptibility and higher tendency for deformation. Another reason for the high stability of the PCR brackets could be the relatively thick bracket base design. The thick base design is necessary to allow individualization to the tooth surface for future clinical use. Nevertheless, this only allows limited comparability of the ceramic and PCR brackets regarding fracture susceptibility.

The possibilities of 3D printing are continuously expanding. It is not surprising that initial trials with in-office printed brackets have already taken place [22–24, 26]. To establish its clinical use, further studies must determine whether the used PCR material really meets the requirements for a bracket material. The possibility of torque transfer to the tooth is of particular research interest, as it represents an essential movement in active orthodontics. Therefore, it was considered critical to investigate the novel high-performance resin in terms of torque stability prior to clinical application.

Limitations of this study mainly concern that in vitro investigations have only limited ability to reproduce clinical conditions. This study provides initial evidence that a self-designed polymer bracket can be a possible alternative to conventional bracket systems. The PCR bracket would give the orthodontist the possibility to program the bracket in a personalized manner and offer individualized therapy, which could improve therapy in combination with a digital set-up [54, 55]. The scope for programming the bracket extends from an individualized bracket base to variations in slot and stem dimensions, to color selection and positioning on the tooth surface. The possible advantages of such an individualized bracket system have yet to be explored more deeply [25]. To reach its full potential, the programming should be based on a 3D data set of the patient’s teeth and bony conditions. In addition, the individualized bracket bases must be positioned precisely with the help of a suitable bonding tray. To enable future clinical use, further studies regarding friction properties, discoloration tendencies, shear bond strength, and positioning accuracy are necessary.

## Conclusions

- The slot accuracy of the in-office manufactured Permanent Crown Resin (PCR) brackets was clinically adequate and compares to established bracket materials.
- The in-office manufactured PCR brackets can withstand a simulated crown torque of up to 60 Nmm and is above the clinically necessary range.
- In addition, in vitro and in vivo studies regarding further material-specific and biomechanical properties are necessary.
